# Physical and Mechanical Properties of Tilapia Scale Hydroxyapatite-Filled High-Density Polyethylene Composites

**DOI:** 10.3390/polym14020251

**Published:** 2022-01-08

**Authors:** C. N. Aiza Jaafar, I. Zainol, M. I. Izyan Khairani, T. T. Dele-Afolabi

**Affiliations:** 1 Department of Mechanical and Manufacturing Engineering, Faculty of Engineering, Universiti Putra Malaysia, Serdang 43000, Malaysia; izyankhairani92@gmail.com (M.I.I.K.); deleafolabitemitope@gmail.com (T.T.D.-A.); 2 Chemistry Department, Faculty of Science and Mathematics, Universiti Pendidikan Sultan Idris, Tanjong Malim 35900, Malaysia; 3 Department of Mechatronics Engineering, College of Engineering and Technology, Achievers University, Owo 341104, Nigeria

**Keywords:** hydroxyapatite, high density polyethylene, composite, silane coupling agent, mechanical properties

## Abstract

The effects of filler loading and silane coupling agent on the properties of hydroxyapatite (HAp)-filled high density polyethylene (HDPE) composites have been studied. The (HAp) powder was successfully produced from tilapia scales using the spray drying process utilized to prepare the HDPE/HAp composites. The FTIR peaks for the untreated HDPE/30HAp composite corresponded to the functional groups of HDPE (C-CH_3_) and –CH_2_ and HAp (PO_4_^−3^ and O-H). The FTIR spectrum for the silane-treated composite showed that the C=O and silanol groups were eliminated, which strongly confirms the chemical interaction between the HAp fillers and the HDPE matrix. The developed composites demonstrated enhanced mechanical performance, and in particular the treated HDPE/30HAp-S composite exhibited superior tensile strength, Young’s modulus and flexural modulus of 28.26 MPa, 1272 MPa and 796 MPa, respectively. In vitro cytotoxicity analysis showed that the developed composites were non-toxic and have great potential to be used for biomedical application.

## 1. Introduction

Biocomposite materials have been used over the years in medicine and dentistry to replace and repair body parts, tissues and organs. These materials have become essential materials in the medical field since they produce no toxic reaction or allergenic symptoms as well as their non-inflammatory property [1]. Natural bone is a composite material comprising a mineral portion which includes little apatite crystals and non-stoichiometric calcium phosphate, a blend which offers exceptional mechanical resistance [2,3]. Pure hydroxyapatite (HAp) is Ca_10_(PO_4_)_6_(OH)_2_ which is the same as the chemical composition exhibited by the HAp present in the hard tissues of teeth and bone in the human body [4]. HAp performs an essential role in different synthetic biomaterials owing to its outstanding biocompatibility [5,6]. Besides enhancing the physical and mechanical strength of these materials, a handful of researchers have studied the potential of HAp in wide-ranging applications including sensors, implants scaffolds and tissue engineering [7].

The high-density polyethylene (HDPE) is a highly reliable class of polyethylene (PE) with average tensile and compressive strengths four times that of the conventional low-density polyethylene (LDPE). HDPE has also been successfully implemented in broad-based engineering applications. Meanwhile, due to its non-toxic and non-staining properties, HDPE has found applications in biomedical engineering, particularly in the preparation of implants and replacement of joints [8,9,10,11]. Moreover, owing to the low bioactivity of synthetic polymers (e.g., HDPE) relative to several other biopolymers such as polylactic acid (PLA), poly(lactic-co-glycolic acid) (PLGA), polycaprolactone (PCL) etc., HAp has been proposed as a reliable reinforcing filler for the polymer matrix due to its remarkable biocompatibility and capability to attach strongly to hard tissues [12].

Even though metal-based implants demonstrate superior strength, their major drawbacks are poor biocompatibility and high density. Adding to that, their relatively superior stiffness promotes stress shielding when employed as implants [13]. For instance, the ranges of the Young’s modulus and ultimate tensile strength of the stainless steel are 190–193 GPa and 515–619 MPa, respectively [14], which are superior to those of human cortical bone (3–30 GPa and 70–230 MPa for modulus and ultimate compressive strength, respectively) [15]. On the other hand, HAp/polymers are better off and can significantly subdue the drawbacks associated with ceramic-based and metal-based implants. Additionally, HAp/polymers can be utilized for the treatment of orbital floor fractures, post-enucleation socket syndrome (PESS), middle ear, bone resorption etc. [16]. Tanner et al. [17] reported the clinical application of HAp/HDPE composites developed by Bonfield et al. [18] as bone implants for over 20 years.

A large fraction of the global population is battling with bone tissue-related challenges including bone cancer, bone fracture, ageing and trauma [19,20]. Therefore, there is considerable need for biomaterials to sufficiently meet global needs. Meanwhile, research is ongoing to unravel more promising and reliable biomaterials for bone tissue replacement. So far, synthetic biomaterials, particularly HAp-based composites, have been employed to replace or revamp bone tissue with a view to improving the patient’s quality of life [21]. Nonetheless, constrained availability and high cost of synthetic HAp are some of the factors limiting the broad utilization of HAp-based composites as implants for bone tissue replacement. Hence it becomes imperative for industrial experts and researchers to place attention on other viable alternatives, particularly from biological wastes that can be converted into resourceful biomaterials like HAp.

HAp extracted from biowaste materials such as animal bone, teeth, coral and eggshell are considered to be readily available, environmentally friendly and economical [22,23,24]. In recent times, HAp biomaterials from fish bone and scales have emerged as the most promising to substitute synthetic and bovine bone HAp in biomedical applications since they are safe and they present low risk of disease transmission [25]. The range of HAp composition in fish scales is between 38–46% with the presence of little percentage of CaCO_3_ content [26]. Besides, several millions of tons of fish scales are generated daily as biowaste materials daily [27], making HAp extraction from fish scale a viable means for the resourceful recycling of this biowaste as well as unlocking additional gains for national economies that rely heavily on seafood exports. Hence, the present study takes into consideration the practicality of this biowaste for medical applications since investigation have further established that the HAp extracted from fish scale has better metabolic activity and more dynamic response to the environment [28].

Meanwhile, the interfacial interaction between the polymer matrix and the HAp filler is one of the important factors controlling the mechanical behaviour of the developed composite. However, achieving the desired interfacial interaction between both materials is a difficult task. Due to this incompatibility problem, surface treatment of the filler using coupling agent has been employed in several investigations to improve the properties of polymer matrix composites [29,30]. Hence, in order to better understand the effectiveness of natural HAp extracted from fish scale, this study aims at systematically investigating the effects of different tilapia scale HAp loading and surface treatment on the mechanical and thermal properties of HDPE/HAp composites as potential biomedical implants.

## 2. Materials and Methods

### 2.1. Materials

The hydroxyapatite (HAp) filler with chemical formula Ca_10_(PO)_6_(OH)_2_) and molecular weight of 502.31 g/mol was supplied by MZ Bio Resources, Hulu Selangor Malaysia in the form of Tilapia fish scale ash obtained from thermal degradation of fish scale at 1200 °C. Commercial HDPE grade CC862 resin was supplied by Innovative Pultrusion Sdn Bhd with density of 0.938 g/cm^3^ and melt flow index (MFI) of 8.0 g/10 min. The commercial 3-methacryloxypropyltrimethoxysilane (CH_10_H_20_O_5_Si) supplied by Mutiara Saintifik Sdn Bhd, Selangor, Malaysia was used as the coupling agent in this study to enhance the interaction between the HDPE matrix and HAp filler.

### 2.2. Processing of Hydroxyapatite (HAp) Powder

The wet ball milling process was performed on the HAp filler using a ball milling machine manufactured by RS Advanced Technology Sdn Bhd, Selangor, Malaysia with specification of 0.5 HP motor, 5 L capacity jar mill and porcelain balls having 28 and 15 mm diameter sizes. The powder to liquid ratio used was 1:4 and milling time was set between 24 h to 72 h. Thereafter, slurry containing HAp powder and deionized water was subjected to the spray-drying process using a spray drying machine manufactured by Agridon Technology Sdn. Bhd, Selangor, Malaysia. The nozzle of the spray dryer sprayed the HAp slurry in the form of fine mist into the hot air in the main chamber. Then, the HAp slurry was constantly stirred using mechanical stirrer to control the homogeneity of the slurry during spray drying process. The slurry mist was evaporated into fine HAp powders and were all collected in the main chamber (MC), secondary chamber 1 (SC1) and secondary chamber 2(SC2) collector. The temperature of the main chamber was fixed at 200 °C to ensure faster heat transfer between product and drying air thus give greatest driving force for water evaporation and the outlet temperature was set at 100 °C. The pressure of the spray drying was set at 5 psi. 

### 2.3. Surface Treatment of Hydroxyapatite (HAp) Powder

HAp loading from the best formulation of composites was surface treated with silane to enhance surface interaction between matrix and fillers. In this study, 30 wt. % HAp was chosen for surface treatment. 1.3% of the coupling agent, 3-methacryloxypropyltrimethoxysilane (MPS) calculated based on surface area of filler and coverage surface area of MPS was diluted in ethanol to make up 50% solution. HAp powders were spread as thin layers and the MPS solution was sprayed layer by layer to ensure uniform surface treatment. Thereafter, the treated HAp was dried at 100 °C for 5 h to give room for complete evaporation of ethanol.

### 2.4. Compounding and Fabrication of Composites

The granules of HDPE were pre-mixed in a ball mill with HAp powder for 1 h to obtain a uniform blend of the composite. Then, the blends of homogeneously mixed HDPE and HAp particles were subjected to melt blending in a twin-screw extruder (DSE) with screw speed of 42 rpm and HAp weight ranging from 10 to 30 wt. % as shown in Table 1. The temperature profile of the extruder was set up between 210 °C and 250 °C at the feed zone, metering zone and die depending upon the volume fraction of HAp particles. The blend was extruded in the form of strands via four holes die with the extruded product passing along a conveyor belt for cooling process. After the extrusion process, the HDPE/HAp composites (filament) were cut into pellets using palletizing machine. Thereafter, the HDPE/HAp composite pellets were moulded into test specimens according to ASTM D3641-15 [31] using an injection moulding machine (Haitian) with tonnage of 150 tonne. The injection temperature was set up at 240 °C with injection time of 6.8 s. The specimens produced from this machine were used for mechanical testing such as tensile, flexural and izod impact test according to the required standard.

### 2.5. Characterization Techniques

#### 2.5.1. Particle Size Analysis

Particle size of HAp was carried out by using Mastersizer 2000 (Malvern Analytical Ltd., Great Malvern, UK). In this study, the particle sizes of HAp powder subjected to different durations of ball milling process (i.e., 24, 48 and 72 h) were measured. After which, the smallest particle size was selected as the HAp filler in the developed composites.

#### 2.5.2. Density Measurement

The density values of the pure HDPE, HDPE/HAp composites and silane-treated HDPE/HAp composite were determined using the electronic densimeter MD-300S. The function of the densimeter is to calculate the specific gravity of the sample.

#### 2.5.3. Fourier Transform Infrared (FTIR)

Fourier transform infrared spectroscopy (FTIR) analysis was conducted using a Nicholet 6700 series equipped with attenuated total reflectance (ATR). Basically, FTIR analysis was used to identify the functional groups in the composites. Composite samples (7 mg) were scraped out and placed on Miracle ATR accessory (miracle base optics assembly). Then, the functional groups and chemical components of all composite samples were identified at a frequency range of 650 and 4000 cm^−1^.

#### 2.5.4. Different Scanning Calorimetry (DSC) Analysis

DSC analysis was conducted on the HDPE, HDPE/30HAp and HDPE/30HAp-S composites samples using DSC 1 Mettler Toledo. Approximately 10 mg of each sample was placed in a pressure-tight cell of the machine. The sample was scanned from room temperature to 600 °C at heating rate of 10 °C/min from under a nitrogen atmosphere with a flow rate of 50 mL/min.

#### 2.5.5. Tensile Test

A tensile test was conducted according to ASTM D638-14 [32] using the universal testing machine (UTM) INSTRON 3366 with a crosshead speed rate of 50 mm/min. Before conducting the test, the thickness and width of the specimen were measured using Mitutoyo thickness gauge and caliper. The gauge length of 50 mm was marked on the narrow section of the specimen and at least five specimens were used to measure the tensile properties of each composition of the HDPE/HAp composites.

#### 2.5.6. Flexural Test

The three-point flexural test was conducted according to the ASTM D790-17 [33] using the INSTRON 3366 machine with a crosshead speed rate of 1.29 mm/min. The specimens were cut into rectangular shapes with dimensions of 3.2 mm × 12.7 mm × 125 mm (thickness × width × length) with support span of 48 mm based on the general rule. Before conducting the flexural test, the thickness and width of the specimen were measured by using Mitutoyo thickness gauge and caliper and at least five specimens were used to measure the flexural properties of each composition of the HDPE/HAp composites.

#### 2.5.7. Impact Test

The izod impact test was conducted on notched specimens according to ASTM D256-10 [34] using CEAST Resil Impactor (MODEL CEAST 6957, serial no 16688, equipment code E74) and a 2 joule hammer. Five notched specimens were tested for each composition and the mean values of the absorbed energy of the composites were computed.

#### 2.5.8. Scanning Electron Microscopy (SEM)

Morphologies of the HDPE/HAp composites fracture surface from tensile and impact tests were examined using a field emission scanning electron microscope (FESEM), Hitachi SI 8020 UHR. The pure HDPE, HDPE/30-HAp and silane treated HDPE/30-HAp composites were selected for the analysis. The surfaces were coated with platinum using Quorum Q150R S to avoid electrostatic charging and to obtain better images for comprehensive analysis where information about the failure mode and the filler distribution can be obtained.

#### 2.5.9. In Vitro Cytotoxicity Test

Cytotoxicity test was carried for untreated and treated HDPE/HAp composites following ISO 10993-5 [35]. The samples were extracted by immersing them in complete media for 24 h at 37 °C without agitation using a weight volume ratio of 200 mg/mL. Pure extracts were then filtered through a 0.2 um syringe filter for sterilization purposes and added to a healthy monolayer L929 cell and later incubated in a CO_2_ incubator for 4 h at 37 °C/5% CO_2_ for 24 h. After 24 h incubation, cell viability was tested using alamar blue assay and the culture was stained with alamar blue solution (1:10) and incubated for 4 h at 37 °C in a CO_2_ incubator. Thereafter, the stained culture then was detected by absorbance at 570 nm using the universal microplate reader.

## 3. Results and Discussion

### 3.1. Particle Size Analysis and Microstructure of HAp Powder

After milling for 24 h, the average particle size (445.977 μm) of fish scale ash (HAp powder) reduced to 2.455 μm as evidenced in Table 2. Upon milling further for 48 h and 72 h, the average particle sizes were 1.859 μm and 2.061 μm, respectively. Clearly, the HAp powder milled for 48 h exhibited the smallest particle size which can be attributed to the attainment of equilibrium in particle breakage during the milling process. However, by extending milling time to 72 h, the particle size of HAp increased. This can be ascribed to particle agglomeration, indicating that the HAp particles started agglomerating after 24 h of milling when the energy required for the breaking of particles had reached its maximum. Hence, the agglomeration of the small particles already formed during the initial periods of milling. The finding is consistent with another investigation [36], where the breakage of particles occurred at the early stage of milling followed by equilibrium between re-agglomeration and de-agglomeration.

**Figure 1:**
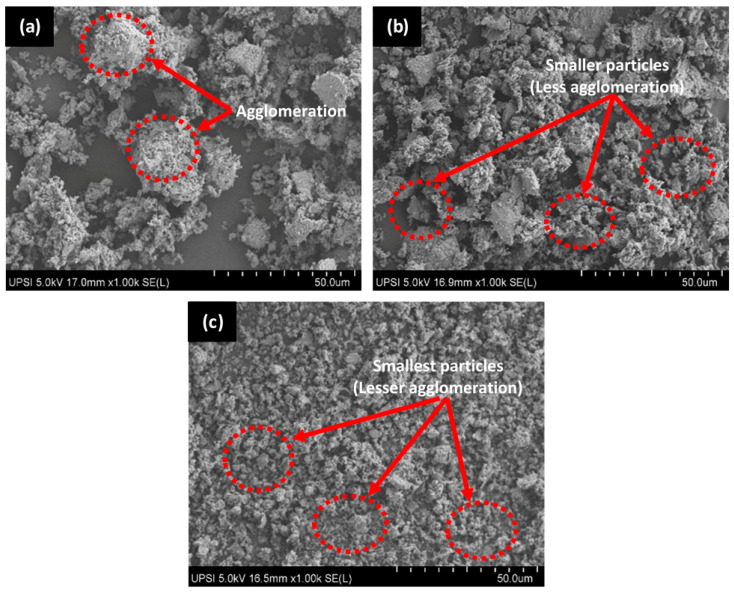
Scanning electron microscopy (SEM) images of HAp powder from (**a**) main chamber (MC) (**b**) secondary chamber (SC1), and (**c**) secondary chamber (SC2).

More so, the particle sizes of HAp powders collected from the spray dryer is summarized in Table 2. As evidenced from the results, particle sizes of HAp differ from one chamber to another which can be attributed to the chamber dimension effect which largely controls the aerodynamics of air/gas flow as well as the resultant residence time of droplets [37]. From Table 2, the HAp collected from the secondary chamber 2 (SC2 = 2.178 μm) exhibited the smallest average particle size followed by the main chamber (MC = 5.674 μm) and the secondary chamber 1 (SC1 = 6.359 μm). The mixture of HAp particles from MC, SC1 and SC2 was used as filler in HDPE/HAp composites which show the mean particle size of 5.180 μm. Figure 1 presents the SEM micrographs of the HAp powders collected from the different chambers of the spray dryer. The microstructures show irregular shape of HAp particles with some degree of agglomeration due to static force between particles, especially for sample from MC, which differs from the more spherical shape of synthetic HAp [38].

### 3.2. Density of Pure High-Density Polyethylene (HDPE) and HDPE/HAp Composites

The measured densities of pure HDPE and HDPE/HAp composites with different filler loading are summarized in Table 3. As evidenced from the table, the density of the composites increased with increasing HAp filler loading where composites loaded with 0, 10, 15, 20 and 30 wt. % HAp exhibited experimental density values of 0.93 g/cm^3^, 1.01 g/cm^3^, 1.05 g/cm^3^, 1.12 g/cm^3^ and 1.18 g/cm^3^, respectively. This trend is expected since HAp is denser than pure HDPE. The density of the HAp used in the current study as measured experimentally is 2.469 g/cm^3^ which is quite close to the density of synthetic HAp (2.93 g/cm^3^) investigated by Smolen et al. [39]. This comparison clearly confirms the viability of HAp extracted from tilapia scale in the development of polymer-matrix composites for biomedical application. More so, the increased densification of the HDPE/HAp composites with rising content of HAp filler was further enhanced by the lower viscosity of the less dense HDPE, which promoted HAp mobility and dispersion in the HDPE matrix. Meanwhile, a negligible difference in density was observed in the silane treated composite (HDPE/30HAp-S = 1.17 g/cm^3^) and untreated composite (HDPE/30HAp = 1.18 g/cm^3^), which is consistent with the finding of Atiqah et al. [40].

### 3.3. Fourier Transform Infrared (FTIR) Spectroscopy of Pure HDPE and HDPE/HAp Composites

**Figure 2:**
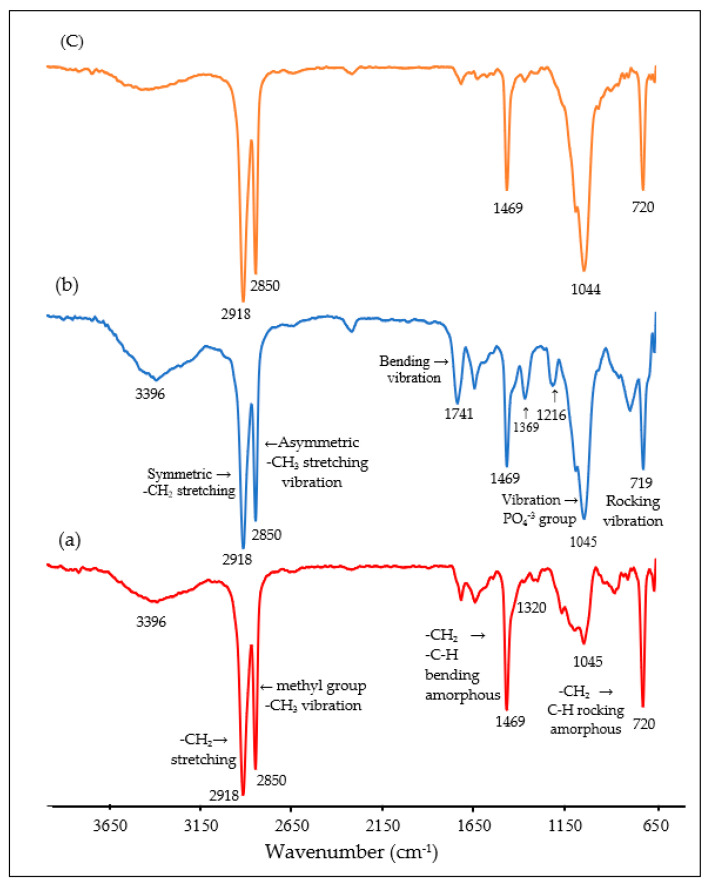
Fourier transform infrared (FTIR) spectra of (a) Pure HDPE, (b) Untreated HDPE/30HAp composite, and (c) Treated HDPE/30HAp-S composite.

Figure 2 shows the FTIR spectra of pure HDPE, HDPE/30HAp and HDPE/30HAp-S composites. As seen from the figure, the characteristic absorption peak at 1469 cm^−1^ for the pure HDPE corresponds to the C-H rocking of amorphous region in HDPE as well as the C–H bending of the HDPE structure. In addition, peaks are observed at 2850 cm^−1^ and 2918 cm^−1^ due to methyl group (C–CH_3_) and –CH_2_ stretching respectively. For the untreated composite the peaks correspond to functional groups of HDPE at 2918, 2850, 1469 and 720 cm-^1,^ extra peaks are observed for HAp component at 1045 cm^−1^ and 1369 cm^−1^ which correspond to PO_4_^−3^ and O-H groups. Extra peaks at 1369 cm^−1^ and 1741 cm^−1^ most probably relate to chemical interactions between the HAp and HDPE matrix.

**Figure 3:**
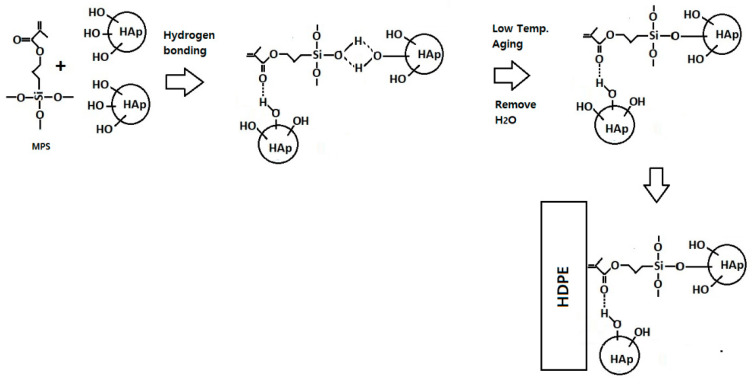
Schematic diagram representing the possible interaction mechanism reaction of silane coupling agent (MPS) interlinked with HAp surface treatment and HDPE matrix.

FTIR spectrum for the silane-treated composite reveals that the peaks at 792 cm^−1^, 1216 cm^−1^, 1369 cm^−1^, 1741 cm^−1^, and 3396 cm^−1^ have been eliminated in the silane treated HAp filler, which strongly confirms the chemical interaction between the HAp fillers, MPS and the HDPE matrix. As established by Liu et al. [41], 3-methacryloxypropyltrimethoxy silane (MPS) can be adsorbed onto the surface of silica from solvent in three possible forms, (1) through the hydrogen bonding between silanol groups of MPS and silica, (2) through hydrogen bonding of C=O and silanol groups, and (3) by forming multilayer in between MPS molecule through hydrogen bonding. The missing infrared absorption frequencies at 1216 cm^−1^ and 1369 cm^−1^ corresponding to C=O stretching can be attributed to the bonding between the O–H and C=O groups from the HAp filler and the MPS silane coupling agent, respectively. More so, a reduction in the intensity of the peak at 3472 cm^−1^ can be observed in the silane-treated composite due to interaction between OH groups of HAp and MPS. Based on the finding by Liu et al. and the FTIR results, the schematic reaction between MPS and surface of HAp particles and then HDPE is shown in Figure 3. Silane coupling agent successfully bonded between HAp fillers and HDPE matrix, thus improved the thermal and mechanical properties. Silane interacted with HAp through formation of covalent bond between OH functional groups in HAp and with silanol on MPS. The methyl groups from MPS interact with HDPE matrix through physical interaction.

### 3.4. Different Scanning Calorimetry (DSC) Analysis

**Figure 4:**
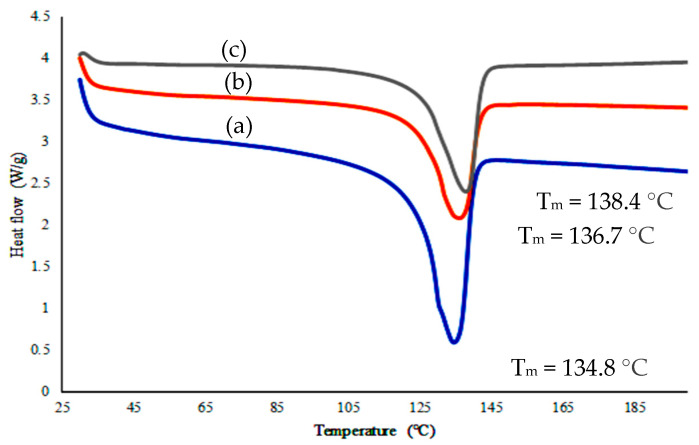
Different scanning calorimetry (DSC) curve of (a) HDPE (b) HDPE/30HAp and (c) HDPE/30HAp-S.

Differential scanning calorimetry (DSC) analyses were carried out to study the influence of filler loading and silane-treated filler on the melting point of HDPE/HAp composite. Figure 4 shows the melting peak of pure HDPE and its composites, evidencing that there is significant variation in their position. The melting point (T_m_) of pure HDPE of 134.8 °C was shifted to 136.7 °C after loading with 30 wt. % HAp. Meanwhile, the melting point of silane-treated composite was shifted to 138.4 °C. This increase could be attributed to the ability of natural HAp in increasing surface interaction between the HDPE matrix and the silane in increasing the HAp–HDPE interfacial bond. This is an agreement with FTIR results which showed spectrum peak changes indicated some degree of chemical interaction between HAp and the matrix.

### 3.5. Tensile Properties

**Figure 5:**
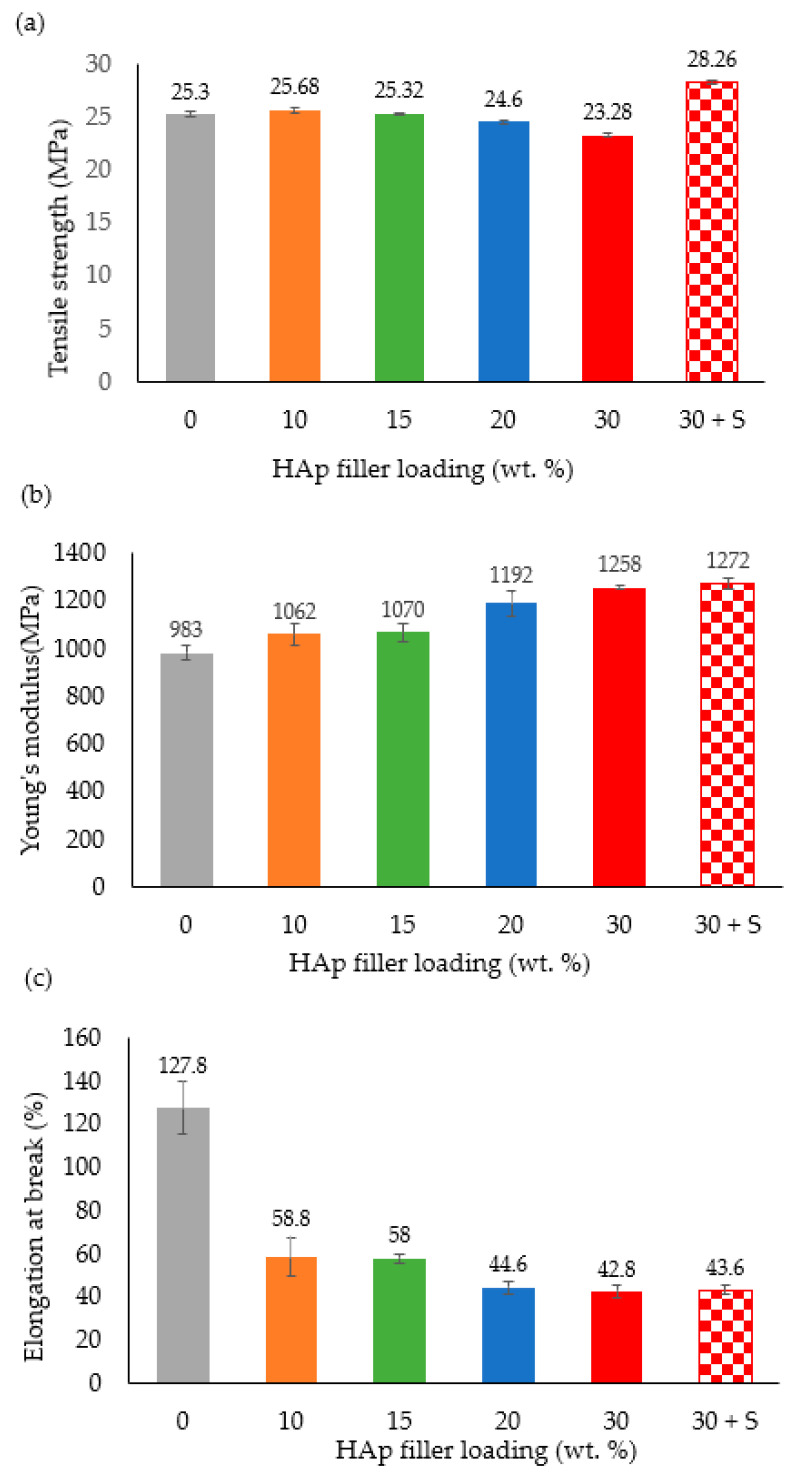
Effects of HAp filler on (**a**) tensile strength, (**b**) Young’s modulus, and (**c**) elongation at break of the HDPE/HAp composites.

Figure 5a presents the effect of HAp filler loading on the tensile strength of HDPE/HAp composites. Obviously, it can be inferred from the figure that the HAp filler enhanced the tensile strength of the composites up to the 15 wt. % mark, which can be attributed to better distribution of the HAp filler in this set of composites. Upon exceeding the 15 wt. % HAp loading, the tensile strength of the composites declined with the HDPE/30HAp exhibiting the lowest tensile strength of 23.28 MPa relative to the pure HDPE (25.3 MPa). The reason behind this sudden decline stems from the HAp loading exceeding the optimal value for effective strengthening of the composites.

Generally, it is believed that the HAp filler tends to agglomerate above its optimal loading, thereby restricting the molecular mobility of the HDPE under stress and increasing the risk of composite failure due to external forces. Moreover, the dysfunctional interfacial bonding between the HAp filler and the HDPE matrix promoted void formation in these composites which resulted in impaired distribution of tensile stresses at the filler–matrix interface. Hence, the tensile strength deterioration observed with increasing HAp filler loading. This observation agrees well with findings reported in some earlier researches [28,42,43].

Contrary to the HDPE/30HAp composite, the silane-treated counterpart (HDPE/30HAp-S) demonstrated the highest tensile strength across the board. The enhancement in the tensile strength of the HDPE/30HAp-S composite can be attributed to the improved bonding between the HAp filler and the HDPE matrix. This improvement led to efficient stress transfer from the matrix to the filler through the filler–matrix interface and concurrent increase in tensile strength. The result can be further substantiated using a complementary study elsewhere [44], where the MPS silane coupling agent increased the wetting and wrapping of the HAp particles in the HDPE matrix. This phenomenon has been confirmed in the SEM analysis presented later in the section.

Figure 5b presents the effect of different HAp filler loadings on the Young’s modulus of the HDPE/HAp composites. As expected, the pure HDPE exhibited the lowest Young’s modulus (983 MPa) owing to higher ductility of HDPE relative to the HAp particles. Generally, the Young’s moduli of the HDPE/HAp composites increased with increasing HAp loading as seen in the Figure. However, a sharp rise in the Young’s modulus can be evidenced beyond the 15 wt. % mark of the HAp loading up to 30 wt. %. This is indicative of the fact that composites loaded with HAp filler below the 15 wt. % mark may not offer high improvement for the Young’s modulus of HDPE. It is also important to note that the silane-treated HDPE/30HAp-S composite exhibited the superior Young’s modulus of 1272 MPa with a corresponding enhancement of about 29.4% relative to the pure HDPE. A similar finding was observed in a complementary study elsewhere [45], wherein silane treatment of fibre-reinforced HDPE significantly enhanced the stiffness of pure HDPE.

Figure 5c shows the plot for the elongation at break of the composites as a function of the HAp filler loading. Clearly from the Figure, the pure HDPE demonstrated the highest ductility with an elongation at break of 127.8%. Meanwhile, the introduction of HAp filler to the HDPE matrix resulted in the immediate transition of the ductile failure exhibited by the pure HDPE to brittle failure in the composites, thereby corroborating the Young’s moduli measured for the composites. Also worth noting is the higher ductility (43.6%) of the HDPE/30HAp-S composite relative to the untreated counterpart (42.8%) which can be explained by the good interfacial adhesion between the silane-treated HAp filler and HDPE matrix as discussed earlier.

**Figure 6:**
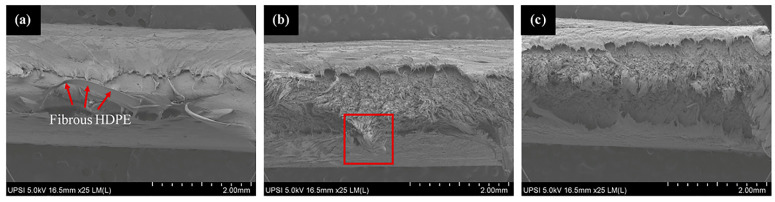
SEM images for the fracture surfaces of the (**a**) pure HDPE, (**b**) HDPE/30HAp, and (**c**) HDPE/30HAp-S composites subjected to tensile test.

The microstructural characterization was performed using the SEM to analyze the fracture behavior of the pure HDPE and HDPE/HAp composites subjected to tensile loading as shown in Figure 6. The pure HDPE as shown in Figure 6a exhibits a ductile fracture behavior with a fibrous appearance of the polymer on the fracture surface (red marked region) which indicates a significant level of plastic deformation common to most polymer materials. Whereas, with the introduction of the HAp filler, the fracture morphology transited to a brittle-type failure (Figure 6b) which supports the relatively high reduction in the elongation at break of the HDPE/HAp composites (Figure 5c). Similar to the the HDPE/30HAp, the silane-treated counterpart also exhibited a brittle failure. However, the fracture surface of the HDPE/30HAp-S exhibits a planar morphology (Figure 6c) relative to the undulating morphology of the HDPE/30HAp counterpart (marked region of Figure 6b), indicating that the silane-treated composite exhibits better filler-matrix adhesion level. Exceptional interfacial bonding offers mechanical interlocking at the filler-matrix interface hence, the superior tensile strength and stiffness demonstrated by the HDPE/30HAp-S composite.

### 3.6. Flexural Properties

**Figure 7:**
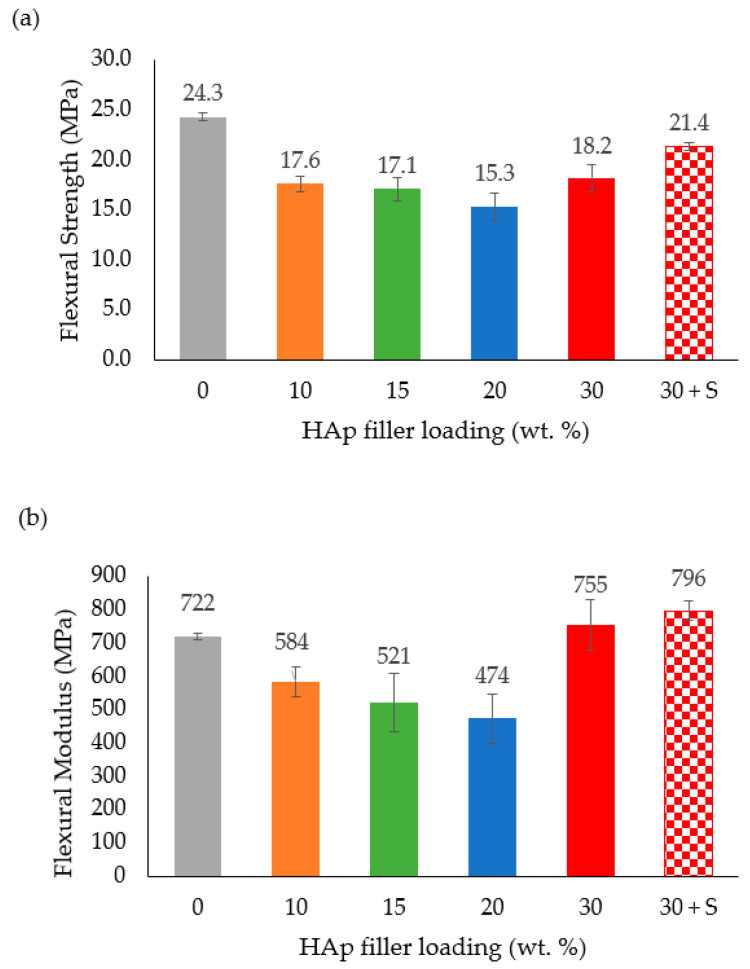
Effect of HAp filler on (**a**) flexural strength and (**b**) flexural modulus of the HDPE/HAp composites.

The flexural strength of the HDPE/HAp composites as a function of HAp filler loading is presented in Figure 7a. It can be observed that the flexural strength decreased linearly with increasing filler loading up to the 20 wt. % HAp mark. The reason for this trend can best be substantiated by the findings of Ohgaki et al. [46] and Damadzadeh et al. [47]. Based on these investigations, flexural strength of the composites declined due to insufficient adhesion of the HAp filler particles to the polymer matrix and, as a result, the stress did not intensify at the HAp–polymer interface. Meanwhile, the HDPE/30HAp-S composite exhibited improved flexural strength as compared to the untreated counterpart which is in agreement with the discussion presented for the tensile properties.

The flexural modulus of the HDPE/HAp composites as a function of HAp filler is shown in Figure 7b. Similar to the flexural strength, the flexural modulus decreased with increasing filler loading up to the 20 wt. % HAp mark. Beyond this mark and as compared with the flexural modulus of the pure HDPE (722 MPa), the HDPE/30HAp and HDPE/30HAp-S composites exhibited higher flexural moduli (and corresponding percentage increases) of 755 MPa (4.6%) and 796 MPa (10.3%). The higher flexural modulus demonstrated by the HDPE/30HAp-S composite relative to the untreated counterpart confirms the capacity of the silane treatment in enhancing the rigidity of the composite. The positive effect of silane treatment on the flexural properties of other polymer-matrix composites has also been widely reported in other researches [48,49].

### 3.7. Impact Energy

**Figure 8:**
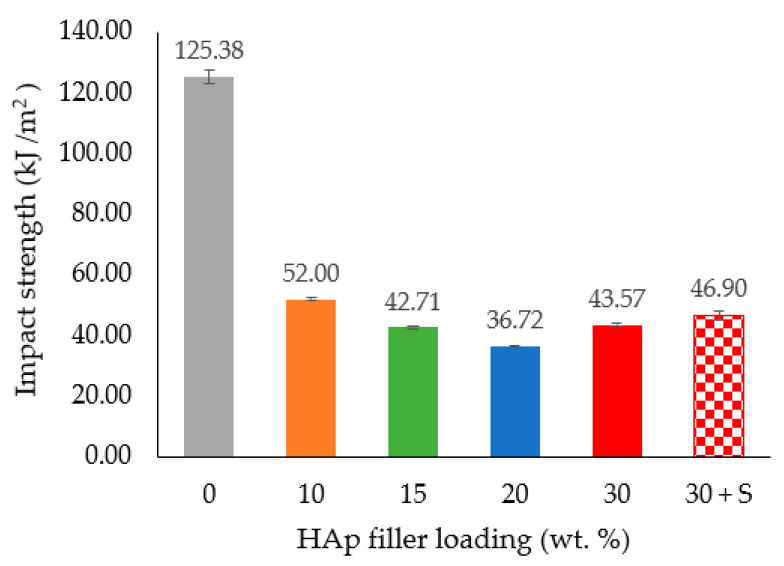
Effect of HAp filler on the impact strength of the HDPE/HAp composites.

The impact strength values for the HDPE/HAp composites at different HAp filler loadings are presented in Figure 8. As seen in the figure, the impact strength dropped drastically from 125.38 KJ/m^2^ for the pure HDPE to 52 KJ/m^2^ after the addition of a small amount (10 wt. %) of HAp filler loading. Moreover, beyond the 20 wt. % mark of the HAp filler loading, the impact strengths of the HDPE/30HAp and HDPE/30HAp-S composites slightly increased to 43.57 KJ/m^2^ and 46.9 KJ/m^2^, respectively. The introduction of hard HAp filler hardened and disrupted the continuity of the HDPE matrix, thus restricting the capacity of the matrix to dissipate the applied impact energy. This submission is consistent with findings presented elsewhere [13] on HDPE/eggshell HAp composites.

**Figure 9:**
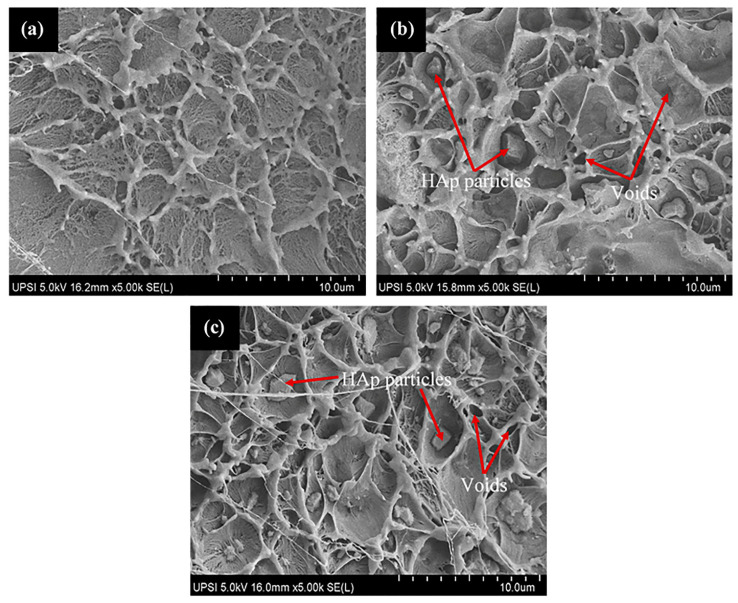
SEM images for the fracture surfaces of the (**a**) pure HDPE, (**b**) HDPE/30HAp, and (**c**) HDPE/30HAp-S composites subjected to impact strength test.

Figure 9 shows SEM images of the impact fracture surfaces of the pure HDPE, HDPE/30HAp and HDPE/30HAp-S composites. As seen in Figure 9a, pure HDPE manifestly exhibits a ductile fracture surface without the presence of HAp filler. However, after adding 30 wt. % HAp filler to the HDPE matrix, agglomeration of HAp particles was detected within the fibrous HDPE polymer in the composite (Figure 9b). This observation confirms the earlier discussion above where the presence of HAp was noted to have triggered disruption in the continuity of the matrix, thereby reducing the capacity of the matrix to dissipate the impact energy. Figure 9c illustrates a relatively homogenous dispersion of HAp particles embedded in the HDPE matrix of the silane-treated composite which accounts for the strength enhancement observed in the mechanical properties except for the impact energy.

### 3.8. Cytotoxicity of HDPE/HAp Composites

**Figure 10:**
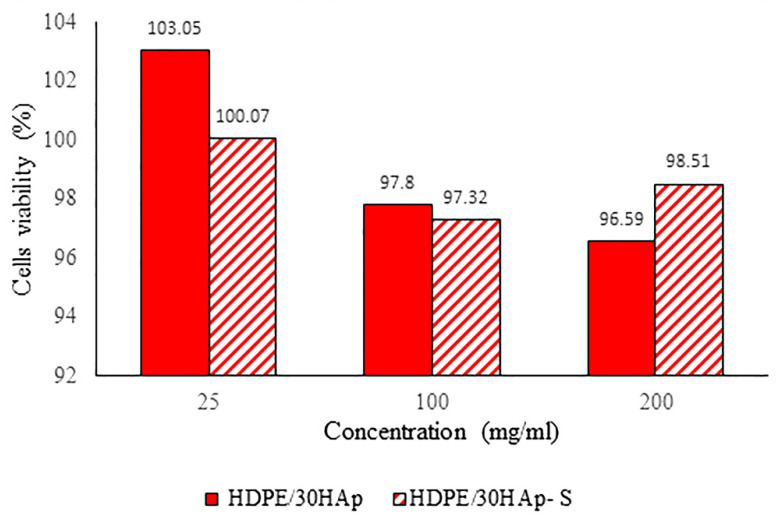
Percentage of cell viability at different concentrations for untreated HDPE/30HAp and treated HDPE/30HAp-S composites.

The result of the cell viability at different concentrations of sample extract is shown in Figure 10. As seen from the figure, the overall results demonstrated that both the treated and untreated HDPE/HAp composites are biocompatible since the cells exposed to 24 h of extract recorded cell viability values higher than 80% for all the tested concentrations. The finding above is consistent with the study of Liu et al. [43] in which it was reported that the cell viability was approximately 100% after 24 h of co-culture, hence indicating excellent biocompatibility of the HDPE/HAp composite. Therefore, it can be concluded that both composites are biocompatible and suitable for biomedical application.

## 4. Conclusions

HAp particles were successfully prepared from tilapia scale using the spray drying process and utilized to prepare the HDPE/HAp composites. Meanwhile, some of the HAp particles were treated with silane in order to investigate the effect of surface treatment on the physical and mechanical properties of the developed composites. The FTIR peaks for the untreated HDPE/30HAp composite corresponded to the functional groups of HDPE (C–CH_3_) and –CH_2_ and HAp (PO_4_^−3^ and O–H). On the other hand, the FTIR spectrum for the silane-treated composite showed that the C=O and silanol groups have been eliminated, which clearly confirms the chemical interaction between the HAp fillers and the HDPE matrix. Experimental results showed that the developed composites demonstrated enhanced mechanical performance, particularly the treated HDPE/30HAp-S composite exhibited superior tensile strength, Young’s modulus and flexural modulus of 28.26 MPa, 1272 MPa and 796 MPa, respectively. In vitro cytotoxicity analysis revealed that both silane treated composite and untreated composites are non-toxic and suitable for biomedical application.

## Figures and Tables

**Table 1:** Composition and designation of high-density polyethylene/hydroxyapatite (HDPE/HAp) composites.

Designation	Composition (wt. %)	
	HDPE	HAp
HDPE	100	0
HDPE/10HAp	90	10
HDPE/15HAp	85	15
HDPE/20HAp	80	20
HDPE/30HAp	70	30
HDPE/30HAp-S (Treated)	70	30

**Table 2:** Particle sizes of HAp powder after milling and spray-drying process.

	Particle Size (µm)		
Milling Time	D_0.5_	D_0.1_	D_0.9_
0 h	445.977	202.928	780.832
24 h	2.455	0.772	7.462
48 h	1.859	0.708	5.919
72 h	2.061	0.854	3.766
**Sources**			
HAp slurry	4.666	0.767	24.717
Main chamber (MC)	5.674	0.829	26.802
Secondary chamber 1 (SC1)	6.359	0.854	20.735
Secondary chamber 2 (SC1)	2.178	0.699	22.629
Mixture (MC + SC1 + SC2)	5.180	0.810	23.343

**Table 3:** Density of the HDPE/HAp composites.

Sample	HAp content (wt. %)	Experimental Density (g/cm^3^)	Theoretical Density (g/cm^3^)
HAp powder		2.469	2.469
HDPE/0HAp	0	0.93	0.93
HDPE/10HAp	10	1.01	1.09
HDPE/15HAp	15	1.05	1.16
HDPE/20HAp	20	1.12	1.24
HDPE/30HAp	30	1.18	1.39
HDPE/30HAp-S	30	1.17	1.39

## Data Availability

The data presented in this study are available on request from the corresponding author.
